# Social Egg Freezing—A Trend or Modern Reality?

**DOI:** 10.3390/jcm13020390

**Published:** 2024-01-10

**Authors:** Dimitra Katsani, Nefeli Paraschou, Eleni Panagouli, Ermioni Tsarna, Theodoros N. Sergentanis, Nikolaos Vlahos, Artemis Tsitsika

**Affiliations:** 1 MSc Program “Strategies of Developmental and Adolescent Health”, School of Medicine, National and Kapodistrian University of Athens, 11527 Athens, Greece; katsanid@gmail.com (D.K.); nefeliparaschou@gmail.com (N.P.); eleni72000@yahoo.gr (E.P.); t.sergentanis@uniwa.gr (T.N.S.); 2 Second Department of Obstetrics and Gynecology, School of Medicine, ‘Aretaieion’ University Hospital, National and Kapodistrian University of Athens, 11528 Athens, Greece; ermioni.tsarna@gmail.com (E.T.); nikosvlahos@med.uoa.gr (N.V.); 3 Department of Public Health Policy, School of Public Health, University of West Attica, 12243 Aigaleo, Greece

**Keywords:** social egg freezing, oocyte cryopreservation, age-related infertility, delayed childbearing, fertility preservation

## Abstract

Introduction: Egg freezing for social reasons is a process in which women who want to preserve their ability to fertilize their own oocytes at an older age freeze their eggs. With the help of in vitro fertilization, the cryopreservation of oocytes for future use is achieved. The aim of this article is to study the reasons, the risks and the effectiveness of the method from a worldwide aspect. Methods: A literature search was conducted to evaluate pertinent studies, using data from the search engines PubMed, Google and UptoDate as well as the medical literature. Results: The reasons for delayed procreation are non-medical, with the lack of an appropriate partner for a family being first on the list. The success rate of this method differs with the age of the woman, the number of fertilized eggs and other factors. Like every medical procedure, this method carries risks that relate to the mother (being of advanced age), the embryo and the procedure of in vitro fertilization. The policies that apply in each country differ depending on respective social, economic, religious and cultural factors. Due to the high cost of the method, its selection remains a choice for only a few, reinforcing social inequality. The question of the medicalization of reproduction remains unanswered in the industry of assisted reproduction. Conclusions: In conclusion, egg freezing for social reasons is gradually becoming more widely known, with the United States of America and Israel being at the top the list. Unfortunately, there is no official data registry, and consequently, no statistical results are yet available for Greece, even though it is a method that more and more women are considering. Nevertheless, there is an imperative need for a universal legal framework for all countries with respect for the needs of every woman and different social conditions. More research and data from the literature are needed in relation to the effectiveness of the method from moral and social perspectives.

## 1. Introduction

Although oocyte cryopreservation was initially used as a fertility preservation strategy for medical indications, currently, it is increasingly used to circumvent age-related infertility [1]. In general, oocyte cryopreservation is a method that has developed in recent years since an increasing number of women have chosen it to preserve their fertility, and it is likely to develop into routine clinical practice [2]. In the UK, a 460% increase in oocyte cryopreservation cycles was reported between 2010 and 2016 [3]. Social egg freezing is directly connected to increasing age in relation to the declining fertility of women. This biological limit is now more crucial because of demographic changes [2]. Women select this method for non-medical reasons such as the absence of a suitable partner for building a family, the development of a career and other social and financial reasons. For example, many countries lack financial programs for the enhancement of fertility and for raising a family, an issue that forces younger women who are not financially stable to postpone the creation of a family. Also, public allowances are limited in many countries in Europe, creating a burden for many women when starting to think of building a family. Although women may experience stress related to finding a partner to have a family with when they are quite young, leading to “panic partnering”, SEF can be an alternative that provides women with greater reproductive autonomy [4]. In general, over the past few years, the mean age at which a woman decides to have her first child has increased to over 30 years of age [5].

The aim of cryopreservation is to delay pregnancy in order to have genetic children later in life. It is considered an act of preventative medicine [6]. Epidemiological studies have shown that women who choose this method are occupationally successful, financially independent and more educated [7]. The purpose of this review is to study the reasons for social egg freezing and to highlight the risks and effectiveness of this method around the world.

## 2. Materials and Methods

### 2.1. Study Design

A literature search was conducted up to 25 September 2023. The method of processing materials was performed alongside the literature review using data from the search engines PubMed, Google and UptoDate, as well as from the medical literature.

A preliminary search was conducted using Google, and a systematic search was then performed using the PubMed database for related articles, meta-analyses and systematic reviews. References of eligible studies and relevant reviews were also searched using a snowballing technique.

Various terms were combined, such as “social egg freezing” OR “oocyte cryopreservation” OR “age-related infertility” OR “fertility preservation”.

### 2.2. Inclusion Criteria

All articles that examined reasons for social egg freezing and highlighted the risks and effectiveness of this method were considered eligible for this review. Articles related to egg freezing for medical reasons were excluded. Regarding study design, cohort studies, cross-sectional studies, case series and case–control studies were considered eligible. No language, gender or other demographic restrictions were imposed. Two authors (D.K. and N.P.) working independently of each other performed the selection of studies.

### 2.3. Data Extraction and Analysis

A piloted data extraction form was used to extract data from the eligible articles, which were reviewed simultaneously and independently by two reviewers (D.K. and N.P.). The following data were extracted for each study: the title of the article, the name of the first author and the year of publication, the region/country in which the survey was conducted, the language, study period, study design, sample size, age range, the selection of the sample, the method of egg freezing used, statistical analyses and the main findings regarding the reasons for egg freezing, and the risks and effectiveness of egg freezing. Any disagreement was resolved by discussion between the reviewers and by team consensus.

## 3. Results

### 3.1. Goals of Cryopreservation

Women who decide to undergo egg freezing do so for various reasons. First of all, their goal is to delay childbearing and, at the same time, maintain their fertility even if they age and their embryos’ risk of aneuploidy increases. SEF provides them with a chance to have genetic children and decreases the risk of stillbirths when egg freezing is performed at a young age [8]. With SEF, women gain a time extension to find the “perfect” partner–father, finish their studies, become financially stable and achieve their professional goals [9]. By freezing their eggs, women aim to combine career and family and consequently increase their reproductive autonomy and social equality [10,11].

### 3.2. Cryopreservation Procedure

First, an initial medical consultation takes place in which the patient–woman discusses with the specialist her medical history and finds a suitable treatment plan for her. A series of blood tests including hormone levels and an ultrasound of the ovaries are performed in order to discuss the final treatment plan. After the patient signs a consent form, she starts to follow a medicinal and injection calendar on the second-third day of her menstrual cycle [7]. At regular time intervals, the doctor checks the ovarian response to the medical treatment using regular blood tests and scans. Thirty-six hours prior to egg collection, a trigger shot (either with h-CG, a GnRH agonist or recombinant human LH) is injected for the eggs to mature [8]. Then, the retrieval of eggs takes place under sedation. The collected eggs are frozen using a process called vitrification and then stored in liquid nitrogen until the woman decides to use them. The procedure of social egg freezing is necessarily followed by in vitro fertilization and an embryo transfer [9,12]. In more detail, the thawed eggs are fertilized with a partner’ s sperm in an IVF laboratory at a later time determined by the woman, and the embryos are transferred to the woman’ s uterine cavity in order to carry out a successful pregnancy [13,14].

### 3.3. Risks Associated with Oocyte Cryopreservation

Current evidence demonstrates that planned oocyte vitrification is a low-risk and safe method of fertility preservation to reduce the risk of age-related infertility, even though fertility cannot be guaranteed [1]. Of course, as a medical procedure, SEF has both short-term and long-term medical risks. The most common short-term risk of egg cryopreservation is oocyte hyperstimulation syndrome, which appears in 5% of stimulation cycles and is classified as mild, moderate or severe. Mild and moderate symptoms appear in 3–6% of the patients presenting with headache, nausea, irritability, chest pain and an increase in body weight. Severe cases appear in 1–3% of patients and can be potentially fatal with symptoms like vomiting, oliguria and thromboembolism [2,8,9,12,14,15] (Table 1).

During oocyte retrieval, some patients experience pleural effusion, pelvic pain, intraperitoneal fluid collection, organ damage and risks associated with anesthesia [8,14,16,17] (Table 1). Also, the bleeding risk increases as more oocytes are retrieved [18] (Table 1).

The long-term risks of SEF are related to IVF, the older age of mothers and risks related to the embryo. Women undergoing IVF can experience risks including preeclampsia, premature birth and IUGR (intrauterine growth restriction) [19,20]. Older pregnant women can develop gestational diabetes mellitus, preeclampsia, ectopic pregnancy and others [21] (Table 2).

The older the woman is at the time of egg cryopreservation, the greater the damage to the spindle apparatus after thawing. Many argue that fertilizing a frozen egg via IVF has lower success rates than doing so with a fresh egg, but this argument is not justified by research [2]. On the contrary, the success rates are approximately the same, considering the fact that existing infertility is treated in the case of IVF without egg freezing [3].

Also, there are risks related to the embryo, like congenital anomalies, congenital heart diseases and carcinogenesis [22,23,24,25], but these are not increased in comparison with children born using fresh eggs [3] (Table 2).

Other medical risks can include perioperative infections or ovarian torsion [26,27].

### 3.4. Cost of Procedure

SEF has a high cost across different countries. The highest cost is reported in the United States of America, whereas the lowest cost is in Mexico, to which many women travel for medical tourism [28,29]. In Canada, the procedure costs CAD 5.000 to CAD 10.000 per cycle and CAD 300 to CAD 500 per year for egg storage. Different policies and costs applied in different regions are summarized in Table 3 [30] (Table 3). In vitro fertilization and oocyte transfer also have high costs. Many report that cost is a limiting factor, with a direct impact on the total number of cycles completed [11]. SEF is not covered by public insurance, and not all women can afford this procedure [16]. Also, there is a lack of financial programs for reinforcing procreation and raising children at a young age [22]. Some private insurance companies have also started to cover the cost of this procedure [31]. In order for the procedure to be cost-effective, studies showed that 49–61% of patients need to return to use their oocytes, which is very high compared to the current rates [3].

### 3.5. Prognostic Factors

The two basic prognostic factors for the successful birth of a fertilized cryopreserved oocyte are the age of the woman at the time of the storage and the number of mature oocytes [32,33]. Current studies show higher success rates for individuals undergoing fertility preservation treatment under age 35 [1]. The ideal age for oocyte freezing is 20 years of age or/and 30–35 years of age. The goal is to retrieve 20 oocytes with the maximum of four cycles of oocyte retrieval. The probability of a live birth derived from a preserved oocyte is 60.5% in women younger than 35 years of age and 29.7% in women over 35 [34]. It is evident that the reproductive success rate depends on the age of the oocytes and not the age of the uterus [3]. The minimum number of oocytes that is needed for a successful pregnancy is 8–10 oocytes [35]. The longer the period of time of expected delay and consequently the older a woman is, the more advantageous cryopreservation is. Women that are 30 years old or younger will probably be able to succeed in becoming pregnant naturally, so there is no such need to cryopreserve oocytes since they will probably not use them. For example, a 42-year-old woman has a 6.6% chance of giving birth with her own fresh oocytes, whereas if she freezes her eggs at the age of 30, she has >40% chance [33].

The mean age that women undergo egg freezing is 38 years. This entails more cycles of hormonal stimulation and higher doses of gonadotropins used. One study with 165 female participants showed that most women considered SEF for 1–2 years before actually undergoing a cycle and that women that underwent multiple cycles were considering freezing their eggs for at least 2 years compared to women who underwent one cycle [5].

Surprisingly, with improvements in medical technology and the wide use of the Internet, since SEF has become popular and more women are interested in the method and the success rates, online calculators are available that can calculate the chance of a live birth based on the age of the woman at the time of cryopreservation and the number of eggs retrieved [36].

Policies differ from country to country depending on social, religious and other factors. In the UK, women are allowed to keep their cryopreserved eggs until the 55th year of age. In Denmark, women over 45 years of age are not allowed to freeze their eggs, restricting the reproductive autonomy of the affected women, and the duration of cryopreservation is strictly 5 years. In Singapore, egg freezing was recently legalized for women 20–35 years old, provided that the eggs are used only when they are married [8,37,38,39,40]. Non-medical egg freezing is a highly controversial issue in Islam, with contradictory fatwas (a kind of religious law) being issued in different Muslim countries. For example, in Malaysia, the basic religious principles are that (i) the extraction of mature egg cells from single women is unacceptable, (ii) the use of egg or sperm cells that were collected before marriage is prohibited and (iii) fertility preservation in anticipation of late marriage is a theoretical scenario [41].

### 3.6. Social Aspects

It is important that a team of specialists is available in order to inform women about the advantages and the disadvantages of the procedure, the possible risks, the duration of cryopreservation, the cost and the expected results of the procedure. There is a need for realistic counseling, particularly with respect to the risk of treatment failure [4]. This team should include a gynecologist/obstetrician, an embryologist and a psychologist [32,42,43]. There should be a detailed discussion with both women and men about their natural fertility burden, the possibilities of IVF, possible risks and success rates [44,45].

According to a questionnaire which asked women to identify their initial source of information about the procedure, most (60.5%) admitted they performed their own research, followed by friends/ family and social media. Only 16% of the women said they were informed by a medical specialist. Women older than 35 years of age admitted they were influenced by medical advice due to age more than women younger than 35 years of age.

Another option for educating women would be for national schools to introduce students to the concept of age-related fertility decline and fertility options.

Emotional support is also crucial since women, especially those that undergo multiple egg-freezing cycles, can experience side effects that interfere with their work and social life during the process of SEF [6].

Especially for men, not much is discussed about the ideal age of the father in regards to parenthood since men become parents at an older age and have a shorter survival period compared to women [46]. This creates the false impression that late pregnancy is an event that can be positively or negatively influenced by the woman alone, focused on the age of the woman and disconnected from socio-economic and psychological factors [3].

### 3.7. Legal Issues and Problems

A question arises when discussing the ownership of stored oocytes. The marital status and gender of the partners should be considered. In some countries like Singapore, only married women are allowed to use their frozen oocytes [8,33,34,35,36]. Concerning same-gender couples, policies differ. As an example, in the UK, the NHS NICE Guidelines suggest that homosexual couples can undergo fertility treatments but primarily refer to IVF treatment by itself. Some regions in the UK, like Wales, fund fertility preservation for transgender couples [47]. Also, in the UK, homosexual couples are allowed to use frozen eggs from an egg donor [48].

The ownership of oocytes after a woman donor’s death is an issue. In most countries like France, Germany and Sweden, it is not legal for the partner to use the frozen oocytes to reproduce with a surrogate mother. In the United Kingdom, the use of sperm/eggs after the death of the donor is allowed for IVF if there is written consent [49].

There are three legal choices regarding the use of oocytes after they have been stored. An insurance contract must be signed stating what will happen if the frozen eggs are not used. The maximum length of egg storage should be clearly stated. The eggs will either be donated to other couples, used for scientific research and experiments or destroyed.

Concerning the storage of oocytes, problems can be encountered like accidents and fires that can cause damage to the oocytes. Consequently, fire security and thermal cameras are often installed to preserve the safety of the oocytes. This indirectly means that the storage cost of eggs increases, as high standards of storage safety are needed. In 2018, a tank failure at the Pacific Fertility Center in San Francisco destroyed about 3500 frozen eggs and embryos. A compensation of 15 million dollars in total was awarded to five patients that lost their eggs or embryos because of this accident for pain, suffering and emotional distress [50].

### 3.8. The Usage Rate of Frozen Oocytes

A study performed at the Brussels Center for Reproductive Medicine concluded that only 7.6% of 563 women that froze their eggs for social reasons between 2009 and 2012 used them for fertilization [34]. Another study of 183 women found that only 6% (11/183) used their oocytes, and only 3 conceived a child [30]. Possible reasons for this could be a preference to conceive naturally and a lack of a suitable partner to create a family with and not wanting to use a sperm donor. In a survey performed in 2012 on 20 Australian patients who underwent SEF, 45% said they would consider using a sperm donor in the future if they could not find a suitable partner. Another survey in Israel in 2012 surveyed 19 women that had frozen their eggs, and 3 of them were considering using donor sperm to help them conceive [28]. A fertility clinic in the UK performed a cross-sectional survey (2008–2018) to which 85 women responded. Out of those who had not used their oocytes (n = 62), 5% were going to use a sperm donor. Fifty-one percent agreed that if they did not eventually find a partner, they would consider using a sperm donor [4]. Finally, the Lister Fertility Clinic in London performed a study between 2011 and 2021 with 191 women that had frozen their eggs for social reasons. Participants that did not have a partner at the time of the SEF cycle, were significantly more likely to agree that they would consider the use of a sperm donor to conceive than were those who had a partner. Similarly, women who underwent SEF during the COVID-19 pandemic were significantly more likely to agree that they would consider using a sperm donor to conceive than were those who underwent SEF prior to the pandemic [31]. An interesting fact from a study showed that women who were older at the time of egg freezing returned sooner to use their eggs compared to those who froze their eggs at a younger age [3].

## 4. Discussion

Social egg freezing is a phenomenon that has been widely broadcast by the media in recent years, creating a state of emotional and psychological pressure for young women to freeze their oocytes in time. The media choose to highlight only the positive aspects of the method without mentioning the possible risks [12].

A qualitative study that was conducted in the in Netherlands in 2021 showed that women who select SEF are driven by feelings of fear, including fear of the lack of an ideal partner and fear of declining fertility. Also, the women in the study followed an “unconventional path to conventional motherhood” in a country like the Netherlands, which is known for its progressiveness and focus on gender equality. It is clear that most women strongly desire a traditional family composition but fear increases when the biological clock is ticking [49].

Also, there are many ethical considerations since issues such as the medicalization of reproduction and its psychological effect on women arise, as many argue that SEF compromises the autonomy of the women involved [51,52]. The medicalization of reproduction refers to the treatment of non-medical reproductive problems as medical within practice and the literature. Sociological research shows that the medicalization of reproduction is directly associated with cultural and social perceptions. Women of reproductive age must cope with varying degrees of social and personal pressures to have children. Because of the pressure they might receive, they are made to consider egg freezing so that that they can have the option to become mothers at a later point in their lives. Evidence shows that women who feel the pressure to have children most intensely are the ones most likely to be excluded from ART (assisted reproductive technology). Also, in many parts of the world, females have lower incomes than their male counterparts, and career pressure often demands that motherhood comes in second place [53].

Another issue being discussed is the age limit of women that want to become pregnant after they have frozen their oocytes. Some authors argue that becoming a mother at an older age can be a problem because of the possible psychological instability of the woman. Nevertheless, older women can be more stable financially and, combined with partnership, can have a stable family environment [3]. Among Western societies, the first-time-motherhood age has increased, while many women of reproductive age underestimate the impact of age on their fertility. In order to avoid the risk of involuntary childlessness, there is a necessity for a further understanding of women’s awareness of their fertility and the options available to preserve it [54]. Age limits for the reproduction of either men or women should comply with the requirements of justice and reproductive equality. It is preferable to make case-by-case decisions rather than have a fixed age limit [3].

The decision regret scale (DRS) was used in a research study in which 201 women participated between 2012 and 2016, showing that 51% did not regret their decision to cryopreserve their oocytes, 33% partially regretted it and 16% strongly regretted it [55]. One research study mentioned that only a small minority of women experience regret after social egg freezing, even if they subsequently achieve live birth spontaneously without using their stored eggs. The women that experienced regret, according to another research study, did so because of the financial expense associated with SEF and the failure to have a child using their frozen eggs. The majority of women who underwent SEF wished they did so earlier [4].

Regarding SEF, in Europe, there is a patchwork of policies regarding this phenomenon. Since there are social, cultural and religious differences from country to country, and considering that SEF is a social issue, it is very hard to achieve universal legislation even though women should be considered equally and have the same rights across Europe. In addition, legal restrictions on IVF and other forms of ART for singles and same-sex couples vary between countries. In a survey of legislation performed in 2014 in 28 EU countries, the use of medical ART by single women was permitted in 11 countries and not allowed in 11 countries, while the legal status was undefined in the remaining 6 countries [50]. Also, the cost of the procedure varies greatly from one country to another [56]. For example, in the UK, the cost of SEF is around GBP 3350, not including medication [57]. On the contrary, the all-in cost (medication, egg freezing and egg storage) in Belgium is slightly lower, between EUR 1500 and 3200 for one cycle [58]. Understandably, oocyte cryopreservation is an expensive procedure and consequently creates social inequalities as it is only available to women that can afford the significant financial outlay. At present, there is a trend that large companies cover the cost of this procedure for their female employees since state insurance systems do not stipulate the costs. For instance, Facebook announced in 2014 that it would cover SEF for its female employees, and other companies soon followed. Interestingly, in recent years, this practice of sponsored egg freezing has often been used by American companies [59,60]. This raises ethical concerns as women are encouraged to be dedicated and productive at work and, at the same time, they are indirectly obliged to postpone motherhood [31].

Questions of whether ARTs (including SEF) are medical procedures at all and if they should be publicly funded or not arise. Some authors argue that ARTs and egg freezing should only be funded if we consider them medically needed treatments. Others, argue that the public funding of ARTs validates, and even intensifies, a socially pervasive pressures to become parents since being free would make it easily accessible to everyone, thereby intensifying social pressures to give birth [53]. On the contrary, considering the fact that until now, SEF has not been covered by public insurance in most regions, the reimbursement of SEF is usually provided only for highly educated women, while their moral stance on the issue of access to SEF strengthens their solidarity with women whose access to the treatment is very limited [61].

An article published in March 2023 discussed the impact of COVID-19 on social egg freezing, and of the women that participated in the study, 44.1% confessed that the pandemic made them more willing to undergo social egg freezing. According to this study, 48.5% of women agreed that COVID-19 restrictions made the SEF procedure SEF more difficult. Furthermore, more than half of the women that participated in the study admitted that “COVID-19 restrictions reduced their chances to meet a partner while socializing” [6].

Even though in many countries like Israel, SEF is a quite common procedure, in Greece, social egg freezing is a practice that became widely known over the last five years, but data are still inadequate for examining the final results. Unfortunately, and to our knowledge, no such records concerning the number of women who choose social egg freezing (for non-medical reasons) are available in Greece. Thus, this seems to be one of the limitations of our study.

## 5. Conclusions

Although social egg freezing is a practice that is becoming increasingly more popular or widespread every year, with the aim of overcoming the biological barrier of age-related infertility, there is still no clear and universal legal legislation for all countries, and there are no data available for the long-term outcomes and consequences of this method. In conclusion, egg freezing for social purposes seems to be rather popular in the United Stated of America and Israel. Further research and analysis are needed to clarify the pregnancy outcomes and the long-term follow up of children conceived using frozen oocytes. Additionally, due to its high economic cost, this method unfortunately remains a good choice for a few, thus reinforcing social inequalities. But the question of the medicalization of reproduction remains open for the assisted reproduction industry.

Finally, there might be a need to review the term “social freezing” as many people claim that it creates social stigma around the procedure and can sound critical as a term.

## Figures and Tables

**Table 1:** Short-term risks of oocyte cryopreservation.

During Stimulation of Oocytes [2,8,9,12,14,15]		During Oocyte Retrieval [8,14,16,17]
Oocyte hyperstimulation syndrome		pelvic pain
Mild and moderate	Severe	intraperitoneal fluid collection
headache	pleural effusion	damage to organs
nausea	vomiting	dangers related to anesthesia
irritability	oliguria	
chest pain	thromboembolic episodes	
increase in body weight		

**Table 2:** Long-term risks of oocyte cryopreservation.

IVF [19,20]	Pregnant Women of Older Age [21]	For the Embryo [22,23,24,25]
Multiple pregnancies	Gestational diabetes mellitus	Congenital anomalies
Preeclampsia	Preeclampsia	Congenital heart diseases
Preterm birth	Preterm birth	Carcinogenesis
Cesarean section	Cesarean section	
SGA/IUGR	Ectopic pregnancy or spontaneous abortion	
	Depends on the general health status of the pregnant woman It increases with older age	

SGA: Small for gestational age, IUGR: Intrauterine Growth Retardation.

**Table 3:** Policies and costs in different regions.

Region	Legal Status	Total Cost (Including Medication and Annual Storage)
Singapore	Allowed for women 20–37 years old but used after being married	USD 7000–15,000
United States of America	Allowed—no age limit or restrictions for length of storage	USD 7000–20,000
Israel	Allowed for women 31–41 years of age	USD 6500–8500
United Kingdom	Allowed—storage for up to 55 years	GBP 4000–4450
Greece	Allowed—storage for a maximum of five years	EUR 3000–4500

## Data Availability

Not applicable.
